# Assessing the risk of birth defects associated with atazanavir exposure in pregnancy

**DOI:** 10.1186/1758-2652-13-S4-P113

**Published:** 2010-11-08

**Authors:** S Esker, J Albano, H Tilson, J Uy, D Arikan, K Krantz, MJ Jiménez-Expósito, D Seekins

**Affiliations:** 1 Bristol-Myers Squibb Research & Development, 777 Scudders Mill Rd, Plainsboro, NJ, USA; 2 Kendle International Inc, Durham, NC, USA; 3 UNC Gillings School of Global Public Health, Chapel Hill, NC, USA; 4 Bristol-Myers Squibb Research & Development, Hopewell, NJ, USA; 5 Bristol-Myers Squibb Research & Development, Paris, France

## Purpose of the study

The Antiretroviral Pregnancy Registry (APR) is an international collaborative operation which utilizes a prospective exposure-registration design to study antiretroviral (ARV) drug exposures during pregnancy. This study assessed the potential for human teratogenic risk of atazanavir (ATV), a protease inhibitor used to treat HIV infection in combination with other ARV agents. ATV exposures are increasingly reported to the APR in this population, reinforcing the need to better understand the risk of birth defects.

## Methods

The analysis population includes all prospectively reported pregnancy exposures with complete exposure and birth outcome data for HIV-infected women enrolled in the APR from January 1, 1989 through January 31, 2010; the ATV subset includes those enrolled since June 2003, when ATV received FDA approval. The prevalence of birth defects after pregnancy ARV exposure is compared both externally, with rates from a population-based surveillance system, and internally between first-trimester and combined second/third-trimester exposures.

## Summary of results

Through January 2010, 698 women with ATV-exposed pregnancies were enrolled. The mean age of these women was 29 years; 12.9% were White, 63.9% Black and 16.7% Hispanic; 87.9% were from the US. 82.5% had a baseline CD4 > 200 cells/mm3. Of the ATV-exposed pregnancies, 588 were eligible for analysis including 567 live births. Among 368 first trimester exposures (167 since 2008), 8 had birth defects (2.2%). The birth defect rate in infants with second/third trimester exposures was 2.5%, and the rate in a non-HIV-infected population (CDC) was 2.72% (95% CI = 2.68-2.76). The risk of defects of first trimester exposures relative to second/third trimester exposures was 0.87 (95% CI = 0.29-2.61). No pattern of birth defects suggestive of a common etiology was observed.

**Table 1:** 

	Exposure to any ARV (Jan 1989-Jan 2010)	Exposure to ATV (Jun 2003–Jan 2010)
Earliest exposure to ARVs		

First Trimester		

•# of defects/live births	127/4563	8/368

•Prevalence (95% CI)	2.8% (2.3%-3.3%)	2.2% (0.9%-4.2%)

Second/Third Trimester		

•# of defects/live births	158/6184	5/199

•Prevalence (95% CI)	2.6% (2.2%-3.0%)	2.5% (0.8%-5.8%)

Any Trimester		

•# of defects/live births	285/10747	13/567

•Prevalence (95% CI)	2.7% (2.3%-3.0%)	2.3% (1.2%-3.9%)

## Conclusions

Prevalence of birth defects among infants prenatally exposed to ATV is not significantly different from internal and external comparison groups. These findings may be useful in counseling patients who are exposed to ATV during pregnancy.

